# Functionalization of the Implant Surface Made of NiTi Shape Memory Alloy

**DOI:** 10.3390/ma16041609

**Published:** 2023-02-15

**Authors:** Karolina Dudek, Tomasz Goryczka, Mateusz Dulski, Bronisław Psiuk, Agnieszka Szurko, Zdzisław Lekston

**Affiliations:** 1 Center of Refractory Materials, Łukasiewicz Research Network—Institute of Ceramics and Building Materials, Toszecka 99, 44-100 Gliwice, Poland; 2 Institute of Materials Science, Silesian Center for Education and Interdisciplinary Research, University of Silesia in Katowice, 75 Pulku Piechoty 1A, 41-500 Chorzow, Poland; 3 The “Edith Stein School with Character” Foundation, Bałtycka 8, 44-100 Gliwice, Poland; 4 A. Chelkowski Institute of Physics, University of Silesia in Katowice, 75 Pulku Piechoty 1A, 41-500 Chorzow, Poland

**Keywords:** electrophoretic deposition (EPD), hydroxyapatite (HAp), NiTi shape memory implant, surface functionalization

## Abstract

To functionalize and improve the biocompatibility of the surface of a medical implant made of NiTi shape memory alloy and used in practice, a clamp, multifunctional layers composed of amorphous TiO_2_ interlayer, and a hydroxyapatite coating were produced. Electrophoresis, as an efficient method of surface modification, resulted in the formation of a uniform coating under a voltage of 60 V and deposition time of 30 s over the entire volume of the implant. The applied heat treatment (800 °C/2 h) let toa dense, crack-free, well-adhered HAp coating with a thickness of ca. 1.5 μm. and a high crack resistance to deformation associated with the induction of the shape memory effect in the in the deformation range similar to the real implant work after implantation. Moreover, the obtained coating featured a hydrophilic (CA = 59.4 ± 0.3°) and high biocompatibility.

## 1. Introduction

NiTi shape memory alloys (SMA), with a chemical composition similar to the equilibrium one, have outstanding properties such as one-way- and two-way-shape memory, superelasticity effects, and acceptable biocompatibility. These properties have made these materials widely used as implants and surgical tools in medicine, orthodontics, soft tissue surgery, cardiovascular applications and, in particular, in orthopaedics [1,2].

In order to improve corrosion resistance and biocompatibility, the surface of NiTi shape memory alloys is modified by applying protective multifunctional ceramics, polymers or composites layers [1,3,4,5,6,7,8,9,10,11,12,13]. However, the best binding of the metallic implant surface with the bone tissue is ensured by covering its surface with a coating composed of calcium phosphates (CaPs). Apatite materials display relatively high biocompatibility, both with hard and soft tissues. Among the various forms of CaPs ceramics, most attention is focused on hydroxyapatite (HAp), β-tricalcium phosphate (β-TCP) and biphasic calcium phosphates (BCP) [14,15,16].

Since the structure of the NiTi alloy, and thus its unique properties, are susceptible to high temperatures, it is crucial to use low-temperature surface modification methods, such as electrophoretic deposition (EPD). EPD provides the opportunity to control coating thickness and homogeneity. Electrophoresis is particularly recommended for forming coatings on substrates with complicated shapes and morphology [17,18,19], such as implants. It is also one of the techniques for producing relatively thick hydroxyapatite coatings [20,21]. However, in the case of NiTi alloys, the unique shape memory effect may be limited by too thick and/or too rigid coatings. Therefore, it is desirable to modify the surface of the alloy by forming thin, micro milimeters layers.

However, in the case of electrophoretic deposition of ceramic layers on a metallic substrate, their heat treatment is necessary. The sintering increases the ceramic coatings’ density and bonding strength to the metallic substrate. According to the literature, hydroxyapatite coatings were subjected to heat treatment at temperatures ranging from 800 °C to 1300 °C for 2 h [18,19,20,21]. On the other hand, at high temperatures, the NiTi substrate is destroyed and loses the unique features of an alloy. Therefore, heat treatment should be carried out at the lowest possible temperature in this case.

Wettability and chemical composition are other crucial factors when shaping the implant surface properties. Surface wettability considerably impacts the absorption of molecules of adhesion-promoting fibroblasts and/or bacteria. It has been confirmed that hydrophile surfaces have better biological activity in contact with bodily fluids, and, as a consequence, they ensure better osseointegration [22].

From the point of view of implant acceptance by the human body and metabolic processes occurring in the surrounding tissues, it is essential also to determine the biocompatibility and check whether the modification causes the desired adhesion of cells and influences their morphology. In the majority of normal body cells, proper adhesion is a basis for processes such as the movement of cells and intercellular communication. It also determines the survival of a cell and its proper physiological function [23]. Adhesive proteins regulate cellular processes, such as recognition of specific receptors, immune response processes (e.g., during inflammation) or apoptosis [24]. Therefore, their malfunction can contribute to several disturbances in the cell’s functioning and, in consequence, affect the human organism’s biological response.

There are few reports in the literature on surface modification of specific medical implants made of NiTi alloy and used in practice. The work is mainly focused on test samples made of sheet metal or wires. The conditions and parameters of surface modification processes of such samples cannot be transferred directly to implants of other, more complex shapes. The present work proposes a way to increase the functionality of a clamp made of NiTi shape memory alloy. The alloy’s surface was functionalized in two stages: passivation and hydroxyapatite (Ca_5_(PO_4_)_3_OH; HAp) coating formation. The paper summarizes the outcomes of morphology, topography, structure, and the functionalized implant’s deformation ability. Moreover, a particular focus was placed on determining the biological response of the hydroxyapatite coating.

## 2. Material Preparation and Methods

### 2.1. Substrate Treatment Procedure

**Figure 1:**
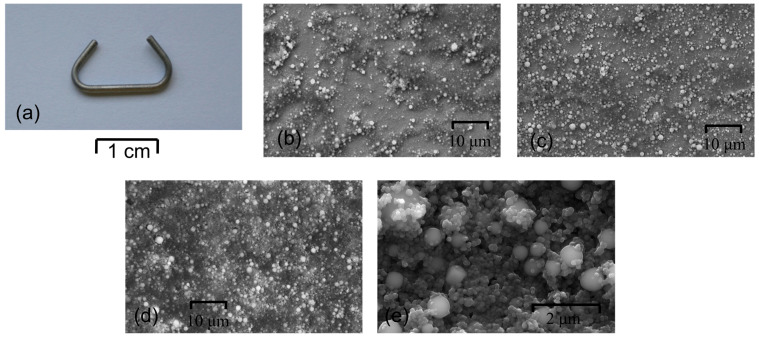
Clamp made of NiTi alloy (**a**) and SEM images of samples coated at: 40 V/30 s (**b**), 50 V/30 s (**c**) and 60 V/30 s (**d**,**e**).

A NiTi clamp with a diameter of 1.2 mm (Figure 1a) and chemical composition of 50.6 at.%. Ni and 49.4 at.% Ti was firstly washed in acetone in an ultrasonic bath to remove trace amounts of impurities. Then, a cleaned clamp was placed in an autoclave at 134 °C for 30 min to form a corrosion-resistive protect thin amorphous TiO_2_ layer on their surface [4].

### 2.2. Suspension Preparation and Electrophoretic Deposition

A colloidal solution was prepared in the form of 0.1 wt.% HAp powder (spherical particles with ca. 100 nm in diameter, structurally with Ca/P = 1.67 and in the crystalline state; Sigma Aldrich, St. Louis, MO, USA) suspended in 99.8% ethanol (Avantor). Then, the as-prepared suspension was used in the electrophoretic deposition (EPD). During the process, a NiTi clamp was a cathode, and platinum was a counter electrode. The deposition was performed at ambient temperature, with voltage reaching 40, 50, and 60 V for 30 s [6]. After deposition, the surface-modified clamp implants were dried at room temperature for 24 h and then sintered in a vacuum furnace at 800 °C for 2 h to increase the coating’s adhesion to the substrate [6].

### 2.3. Coating Characterization

An X’PertPro MPD PANalytical X-ray diffractometer and grazing incidence X-ray diffraction technique (GIXD) with CuKα radiation were used to determine the structural properties of the substrate and coat-forming material. The GIXD patterns were measured at a constant incidence angle of 0.3° at room temperature. A confocal Raman spectrometer WITec alpha 300 R equipped with a laser (λ = 532 nm, P = 40 mW) and a high sensitivity back-illuminated Newton-CCD camera was applied to local structural investigation of the coat-forming materials. As a result, Raman imaging was carried out in a 1600 µm^2^ area, taking into account 25,600 pixels (=spectra) and an integration time of 60 ms per spectrum. All the spectra were collected at room temperature using Olympus objective (100×/0.9 NA), a lateral resolution of 3 cm^−1^, precision of 1 cm^−1^, and a 120–4000 cm^−1^ range. The data in post-processing analysis were subjected to cosmic ray removal and baseline correction using WITec Project Four Plus (version 4.1, WITec Wissenschaftliche Instrumente und Technologie GmbH, Ulm, Germany). A Lorentz–Gauss function is applied to band fitting using the Grams (version 9.2, Thermo Fisher Scientific, Waltham, MA, USA) software package.

Before and after the deposition, the materials’ morphology was visualized using a JEOL JSM-6480 and a TESCAN Mira 3 LMU scanning electron microscope (SEM). Samples for the NiTi/coating cross-section SEM observations were embedded in graphite resin and polished with 2000-grit SiC paper, 1-µm diamond suspension, and finally, 0.1-μm colloidal silica suspension, to achieve a mirror finish. Observations were carried out for samples covered with a 5 nm Cr layer using Quorum Q150T ES equipment. The deformation ability of the HAp coating was observed using a scanning electron microscope during the constant clamp deformation. The implant was first immersed in liquid nitrogen to induce the martensitic transformation and, next, subjected to deformation. After the implant returned to the initial shape at room temperature, the procedure was repeated. Measurements of water contact angle were taken with an OCA 15EC goniometer, with accuracy reaching ±0.01°, by the sitting drop method. Ten images of water drop having a volume of ca 5 µL, placed on the examined surface, were recorded for 10 s. The average contact angle (CA) values were calculated based on the obtained images. The contact angle final value was assumed to be the average of three measurements taken in different parts of the examined surface.

### 2.4. Biocompatibility Studies

Biocompatibility studies were performed on normal human dermal fibroblasts (NHDF) cells after 24, 48, and 72 h. Cytotoxicity was spectrophotometrically measured at absorbance maximum (λ = 490 nm) using an SYNERGY4 multi-dish plate reader (produced by BioTek Instruments, Winooski, VA, USA). The morphology and adhesion of NHDF cells were observed after each 24 h of NHDF growth using a fluorescence microscope (Olympus IX81). The MTS method tested the toxicity of the deposited HAp coating against normal human dermal fibroblasts. The method involves determining the mitochondrial dehydrogenase activity level. The number of viable and metabolically active cells was determined using the MTS assay.

## 3. Results and Discussion

Before the EPD of hydroxyapatite, the NiTi implant surface was passivated in an autoclave. As a result of autoclaving, a self-passivating amorphous TiO_2_ layer was obtained on the surface of the NiTi alloy, which primarily improves the corrosion resistance of the alloy [8,25,26,27,28]. The layer of titanium oxides reached a thickness of approx. 3.5 nm, shows good adhesion to the metallic substrate, is homogeneous along the entire length and has the ability to be used in dynamic conditions [6,27,28]. The implant’s surface was next modified by the deposition of hydroxyapatite using electrophoretic deposition method (EPD) method.

Microscopic observations showed the influence of the applied deposition parameters on the layers’ quality. The morphology of HAp coatings deposited on the passivated NiTi clamp is presented in Figure 1b–d. The deposition process carried out at 40 V and 50 V for 30 s did not provide uniform coverage of the implant surface. HAp particles formed agglomerates, and the surface between them remained unmodified (Figure 1b,c). In turn, the use of 60 V and 30 s resulted in the formation of a most promising homogeneous coating on the entire surface of the clamp (Figure 1d,e). Larger spherical particles were distributed within the tinier hydroxyapatite ones. In such a coating, larger HAp particles should imply an increase in coating diversity and roughness.

**Figure 2:**
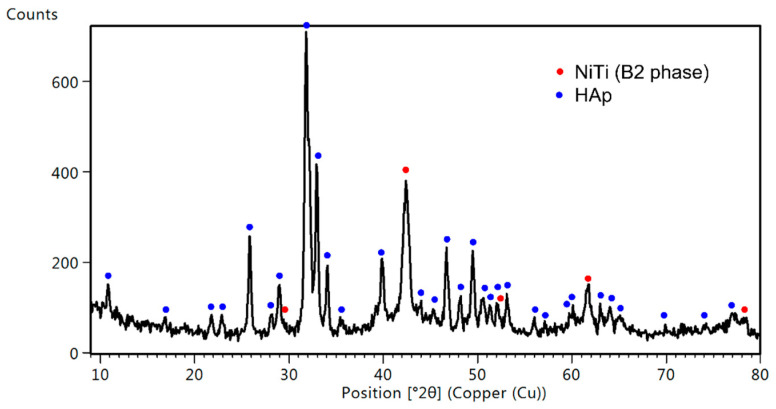
XRD pattern measured at the incidence angle of 0.3° for the sample coated at 60 V for 30 s.

Phase identification (Figure 2) for the coating electrophoretically formed at 60 V and 30 s proved the formation of hydroxyapatite Ca_5_(PO_4_)_3_OH with a hexagonal crystal system (P6_3_/m). Diffraction lines belonging to the B2 parent phase of the NiTi alloy with cubic symmetry (Fd-3m) were also identified.

At a subsequent stage, heat treatment was applied to increase the ceramic coatings’ density and their bonding strength to the NiTi substrate. According to the literature, electrophoretically-formed coatings require sintering, which for HAp ranges from 800 °C to 1300 °C for 2 h [17,18,19,20,21,29,30,31]. However, high-temperature annealing causes shrinkage of the ceramic coating. Therefore, the samples were vacuum-sintered at a lowest reported temperature of 800 °C for 2 h. As reported in [4], the bonding strength between the electrophoretically deposited HAp coating on the passivated NiTi substrate and the one sintered in vacuum at 800 °C for 2 h, as measured by a shear strength test, was found to be (15.1 ± 1.4) MPa. It accounts for about 40% of the shear strength of the cortical bone (34 MPa) [32,33].

**Figure 3:**
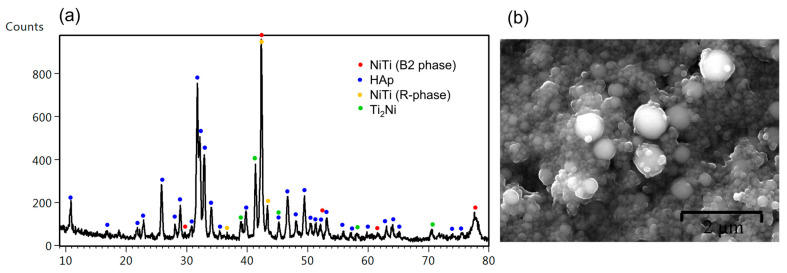
XRD patterns measured at the incidence angle of 0.3° (**a**) and SEM image of the HAp layer deposited at 60 V/30 s after sintering at 800 °C/2 h (**b**).

XRD measurements revealed that the applied heat treatment did not provide for the decomposition of the HAp. However, new phases in the NiTi substrate were created. The B2 parent phase of NiTi alloy was partially transformed to R-phase with a rhombohedral lattice (P-3), and the equilibrium Ti_2_Ni with a cubic lattice (Fd-3m) was precipitated. The most important result of the heat treatment was the sintering and consolidation of HAp particles. In Figure 3b, there are visible spherical forms of HAp with variable sizes and numerous direct bonds characteristic of a material with a high degree of sintering. The fine fraction is much more reactive due to the greater development of the specific surface compared to particles of a larger size, which causes them to sinter first. The applied heat treatment temperature was close to the hydroxyapatite’s dehydroxylation temperature [34], which significantly facilitated the sintering process. Moreover, no cracks induced by high-temperature impact were found.

Raman imaging was performed to shed more light on the spatial diversity of the coat-forming material of a particular volume (globally) and obtain more details in the form of individual analysis (locally).

**Figure 4:**
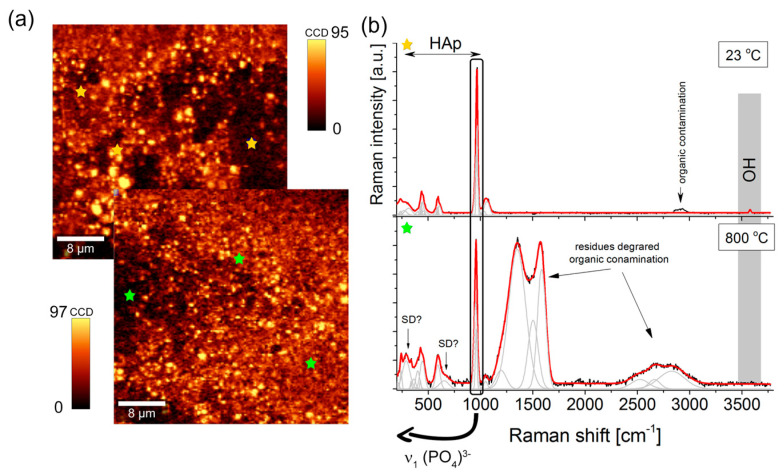
(**a**) 2D Raman imaging map of the coating before (upper panel) and after (bottom panel) heat treatment obtained on the basis of an integrated intensity analysis of the band in the 1000–900 cm^−1^ (ν_1_(PO_4_)^3−^) range. (**b**) Raman spectra collected on the HAp coating deposited at 40 V/120 s before and after sintering at 800 °C for 2 h. Yellow and green stars represent exemplary places from which Raman spectra were exported.

Raman imaging analysis with integration over the 1000–900 cm^−1^ region originating from the symmetric ν_1_(PO_4_)^3−^ modes of HAp visualized a homogenous distribution of brown and yellow spots (Figure 4a). A brighter signal resulted from hydroxyapatite agglomerates with the size of a few microns. Nevertheless, the chemical image corresponded very well with SEM observation and electrophoretic assumptions and confirmed HAp distribution around the entire volume of the coating. On the other hand, the sintering process modifies the coat-forming materials., including surface morphology, insignificantly. The Raman signal and band intensity of the ν_1_(PO_4_)^3−^ mode of HAp (number of counts close to ~100 CCD) in comparison with the analyzed surface before and after annealing found on the same level points to lack of hydroxyapatite decomposition (Figure 4b).

A more detailed analysis of the averaged Raman spectra of many yellow spots provides a typical hydroxyapatite band arrangement with bands at 970 and 961 cm^−1^. Their position and full width at half maximum confirmed the stoichiometric nature of HAp. The remaining bands in 1190–1020, 635–560, and 450–400 cm^−1^ can be ascribed to asymmetric and deformational vibrations (ν_3_, ν_2_, ν_4_ modes) of (PO_4_)^3−^. Low frequency bands ranging from 350 to 150 cm^−1^ originated from vibrations of entire molecular fragments within Ca(PO_4_), including O-Ca-O and O-P-O motions. On the other hand, the high-frequency region was determined by low-intensity bands around 2900 cm^−1^, proving the presence of organic contaminations from the initial material production process, and low-intensity bands around 3578 cm^−1^, characteristic of OH group vibrations [34,35,36,37,38,39].

The average Raman spectrum of the sintered sample is closely related to that of unheated NiTi alloys. The prominent HAp bands remain unchanged, while in the fingerprint region, intense and broad bands of the 1550–1280 cm^−1^ region correlated with the carbon due to the thermal decomposition of carbon-origin contaminants. Carbon incorporated into the hydroxyapatite structure isomorphically substituted OH groups primarily. This process occurred due to high-temperature conditions that induced HAp structure defects (Figure 4b).

**Figure 5:**
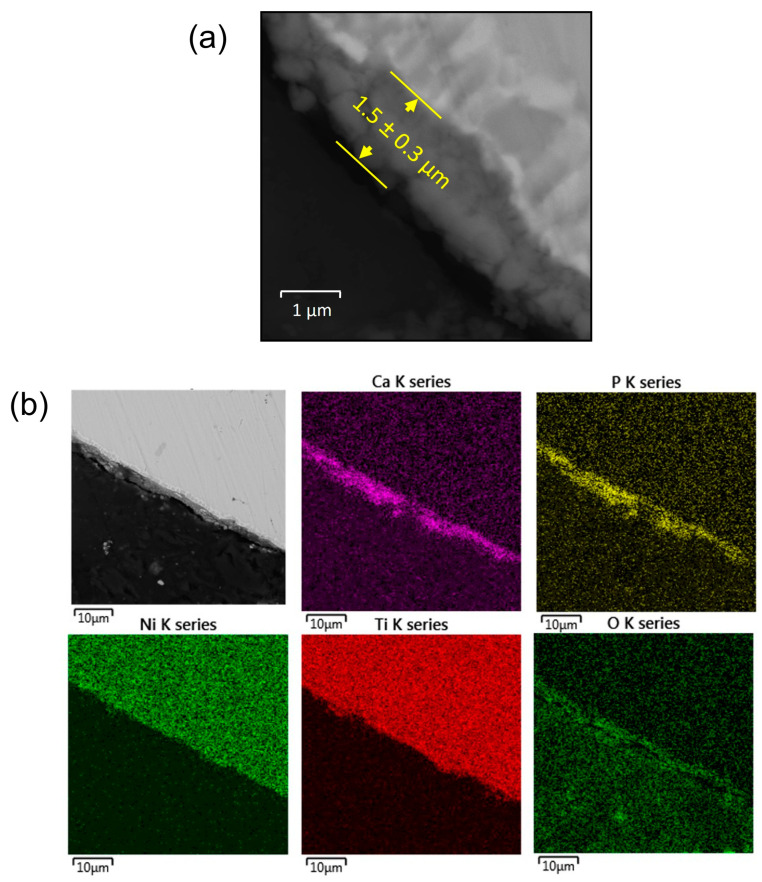
Cross-sectional SEM image (**a**) and distribution map of elements from the selected area (**b**) for the sample after heat treatment.

Observations on the cross-sections (Figure 5) revealed that the layer was 1.5 μm thin. SEM images of the analyzed area supported by Ca, P and O distribution maps showed that the coating adhered very well to the NiTi substrate, without delamination, and homogenously covered the entire surface of the clamp (Figure 5a). It is worth noting that the unique shape memory effect may be limited or entirely blocked by too thick and/or too rigid coatings. Moreover, such coatings cannot deform within a range similar to the shape memory effects. According to the literature, the resistance to crack and deformation of a HAp layer obtained using EPD which was ca. 2.4 μm thick reached 2.48%, and no layer spalling was found [4], even at the maximum deformation value ε = 3.53%. The coating formed on the clamp is thinner, which allows us to assume that it will have an even better deformation ability associated with the shape memory effect.

**Figure 6:**
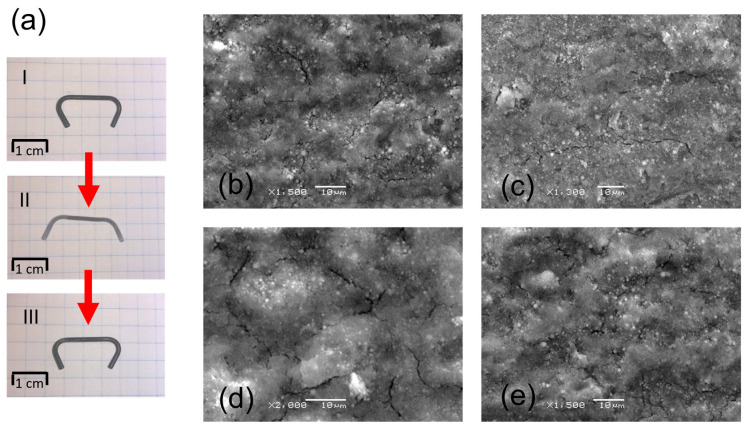
Clamp deformation (**a**) and SEM images of the coated clamp surface after the first deformation cycle (**b**,**c**) and after the second cycle (**d**,**e**). The surface under tension (**b**,**d**) and compression (**c**,**e**).

Thus, the obtained layers were characterized by deformation resistance associated with the induction of the shape memory effect in the implant (Figure 6a). The deformation range corresponds to the deformation the implant is subjected to during implantation. First, the clamp was immersed in liquid nitrogen, and then, after the martensitic transformation, it was deformed to the form shown in Figure 6a, stage II. After reaching room temperature, due to the reversible martensitic transformation, the implant returned to its original shape (Figure 6a, stage III). This proves that there was no plastic deformation of the material and that the applied heat treatment did not reduce the shape memory effect. In this process, part of the clamp was subjected to tensile forces and other parts to compression. Microscopic observations after one and two deformation cycles revealed that neither tensile nor compression stresses caused layer delamination or detachment of individual HAp particles from each other. After the second deformation cycle (Figure 6d,e), only crack propagation in the layer was observed.

**Figure 7:**
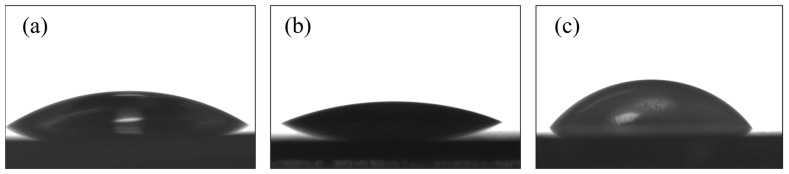
Image of a drop of water on the surface of NiTi alloy (**a**), a layer after deposition (**b**) and after sintering (**c**).

When shaping implant surface properties, one should also consider the wettability of the surface, which influences their biological activity in the bodily fluid environment and provides better osseointegration. Wettability of the hydroxyapatite layers revealed that the contact angle (CA) measured for the deposited coating (Figure 7b), and for the coating subjected to sintering at 800 °C for 2 h (Figure 7c), reached the following values: (25.3 ± 0.4)° and (59.4 ± 0.3)°, respectively. The hydrophilicity of the surface is affected by various functional groups and the topography of the surface. As a result of the applied heat treatment, some structural changes occurred in the hydroxyapatite layer. One is incorporating carbon into the hydroxyapatite structure, isomorphically substituted OH groups, and as a consequence, forming carbonate apatite. According to ref. [40], this process was related to the partial dehydroxylation of hydroxyapatite. The change in hydrophilicity may also be influenced by a change in the surface topography caused by the sintering of the ceramic particles. However, the obtained results confirmed the beneficial hydrophile nature of the formed hydroxyapatite layers. The NiTi shape memory alloy also showed hydrophilic properties compared to coatings. The contact angle showed values higher than the hydroxyapatite layer after deposition but lower than after heat treatment, and reached a value of (33.4 ± 0.5)°.

**Figure 8:**
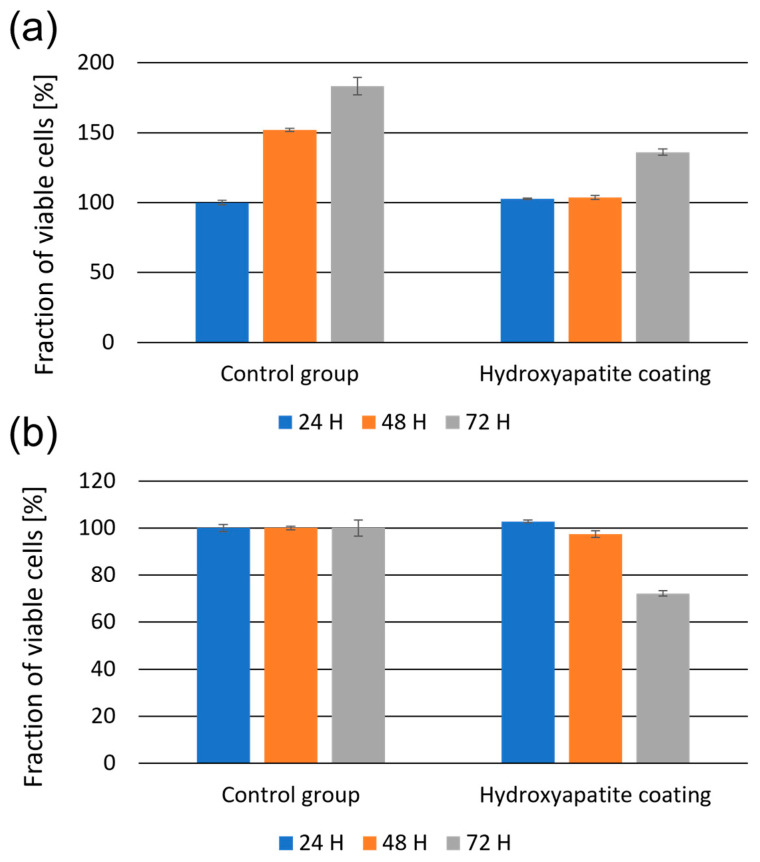
The fraction of viable NHDF cells growing on standard Petri dishes and on the hydroxyapatite coating assessed by the MTS assay. The results were calculated on the basis of control cells, which were grown for 24 h on a standard Petri dish, (**a**) and on the basis of a comparison between cells growing on the substrate with corresponding cells from the Petri dish at each of the tested time points (**b**).

Another critical aspect in the case of implants is their biocompatibility. The results of the MTS assay presented in relation to the control cells growing for 24 h on a standard Petri dish were treated as 100%. This was separate from the control group and from the hydroxyapatite coating (Figure 8a). The number of cells growing for 72 h on a standard Petri dish increased gradually to 180%, while cells growing on the hydroxyapatite coating reached a new confluence of 40% less than the control cells. After 48 h, the enzyme level was still comparable to that at the time of seeding, then after 72 h was around 145%. The alternative approach calculates the abundance of cells growing on the substrate in relation to corresponding cells from the Petri dish (control group) at each of the tested time points (Figure 8b). With the time increase, the cells were observed to stop dividing as intensely as in the control group. On the hydroxyapatite coating, 100% of living cells were maintained relative to the control group for 48 h. Then, after 72 h, the inhibition of cells growth decreased to ca. 70%.

**Figure 9:**
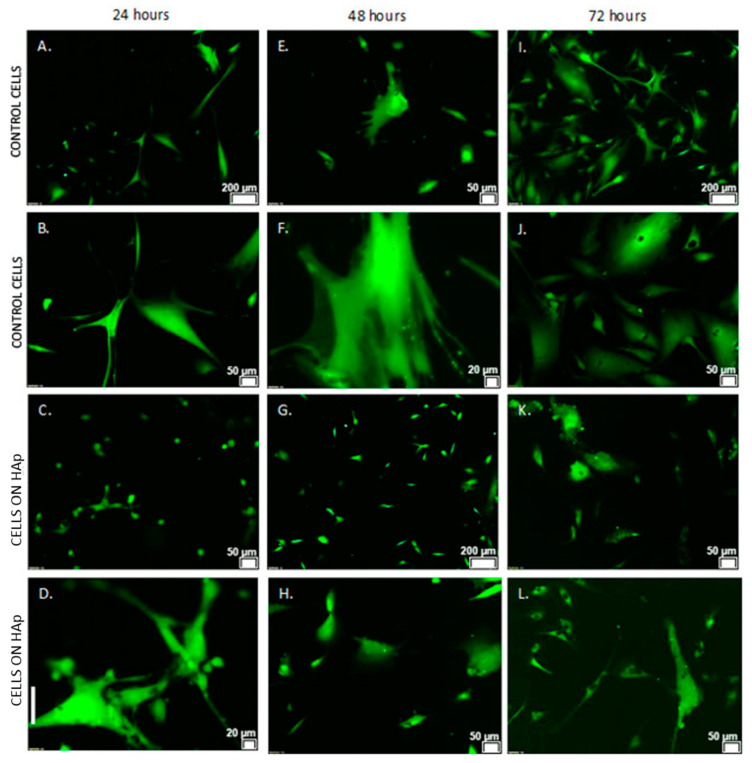
Morphology of control NHDF cells grown on standard Petri dishes ((**A**) magnification 4×, (**B**) 10×) and on the HAp surface ((**C**) magnification 10×, (**D**) 20×) for 24 h; ((**E**) magnification 4×, (**F**) 20×) and ((**G**) magnification 4×, (**H**) 10×) for 48 h; ((**I**) magnification 4×, (**J**) 10×) and ((**K**) magnification 10×, (**L**) 10×) for 72 h.

The morphology and adhesion of NHDF cells were verified by the settlement of cells on a modified clamp substrate. Outcomes were analyzed with a fluorescence microscope at subsequent stages of the test.. Both the NHDF cells on Petri dishes (control sample) and those on the hydroxyapatite coatings showed that even after 24 h, the majority of cells had a well-developed morphology and were well adhered to the surface (Figure 9). The degree of their surface coverage increased with time extension and was comparable in both groups.

## 4. Conclusions

The paper summarized the approach to hydroxyapatite (HAp) Ca_5_(PO_3_)OH coating formation on the surface of a passivated implant, a clamp made of NiTi shape memory alloy. Electrophoretic deposition from the colloidal suspension 0.1% wt. HAp powder in 99.8% ethanol at a voltage of 60 V for 30 s resulted in a thin homogeneous coating covering the implant’s entire surface. The applied heat treatment (800 °C/2 h) allowed to obtain a dense and crack-free HAp coating with a thickness of ca. 1.5 μm. The heat treatment process did not change the structure of the hydroxyapatite coating. However, an essential feature of the coating is its ability to follow shape changes caused by shape memory effects. The obtained layer revealed crack resistance to deformation associated with the induction of the shape memory effect in the implant. Neither tensile nor compression stresses caused layer delamination. The cells growing on the layer were characterized by high mitochondrial enzyme activity (the percentage of viable cells was the same as in the control group for 48 h, and slightly lower after 72 h) and excellent adhesion to the substrate.

## Data Availability

Not applicable.

## References

[1] Yoneyama T., Miyazaki S. (2008). Shape Memory Alloys for Biomedical Applications.

[2] Wadood A. (2016). Brief Overview on Nitinol as Biomaterial. Adv. Mater. Sci. Eng..

[3] Aksoy M.E., Aksakal B., Aslan N., Dikici B. (2021). Boron-Doped Hydroxyapatite Coatings on NiTi Alloys Using the Electrophoretic Deposition Method: Enhanced Corrosion and Adhesion Performances. J. Mater. Eng. Perform..

[4] Dudek K., Goryczka T. (2016). Electrophoretic Deposition and Characterization of Thin Hydroxyapatite Coatings Formed on the Surface of NiTi Shape Memory Alloy. Ceram. Int..

[5] Maleki-Ghaleh H., Khalil-Allafi J., Khalili V., Shakeri M.S., Javidi M. (2014). Effect of Hydroxyapatite Coating Fabricated by Electrophoretic Deposition Method on Corrosion Behavior and Nickel Release of NiTi Shape Memory Alloy. Mater. Corros..

[6] Dudek K., Plawecki M., Dulski M., Kubacki J. (2015). Multifunctional Layers Formation on the Surface of NiTi SMA during β-Tricalcium Phosphate Deposition. Mater. Lett..

[7] Sun F., Pang X., Zhitomirsky I. (2009). Electrophoretic Deposition of Composite Hydroxyapatite–Chitosan–Heparin Coatings. J. Mater. Process. Technol..

[8] Freitag M., Łosiewicz B., Goryczka T., Lelątko J. (2012). Application of EIS to study the corrosion resistance of passivated NiTi shape memory alloy in simulated body fluid. Solid. State Phenom..

[9] Nazarov D., Rudakova A., Borisov E., Popovich A. (2021). Surface Modification of Additively Manufactured Nitinol by Wet Chemical Etching. Materials.

[10] Wang H., Kalchev Y., Wang H., Yan K., Gurevich E., Ostendorf A. (2020). Surface modification of NiTi alloy by ultrashort pulsed laser shock peening. Surf. Coat. Technol..

[11] Safavi M.S., Bordbar-Khiabani A., Walsh F.C., Mozafari M., Khalil-Allafi J. (2023). Surface modified NiTi smart biomaterials: Surface engineering and biological compatibility. Curr. Opin. Biomed. Eng..

[12] Dunne C.F., Roche K., Ruddy M., Doherty K.A.J., Twomey B., O’Donoghue J., Hodgson D., Stanton K.T. (2018). Deposition of Hydroxyapatite Onto Superelastic Nitinol Using an Ambient Temperature Blast Coating Process. Shap. Mem. Superelasticity.

[13] Fathyunes L., Sheykholeslami S.O.R. (2020). The surface modification of Nitinol superelastic alloy with alkaline-heat treatment and hydroxyapatite/chitosan composite coating for biomedical applications. J. Ultrafine Grained. Nanostruct. Mater..

[14] Dorozhkin S.V. (2015). Calcium Orthophosphate Deposits: Preparation, Properties and Biomedical Applications. Mater. Sci. Eng. C.

[15] Dorozhkin S.V. (2012). Calcium Orthophosphate Coatings, Films and Layers. Prog. Biomater..

[16] Dorozhkin S.V. (2013). Calcium Orthophosphate-Based Bioceramics. Materials.

[17] Ferrari B., Moreno R. (2010). EPD Kinetics: A Review. J. Eur. Ceram. Soc..

[18] Boccaccini A.R., Keim S., Ma R., Li Y., Zhitomirsky I. (2010). Electrophoretic Deposition of Biomaterials. J. R. Soc. Interface.

[19] Zhitomirsky I. (2002). Cathodic Electrodeposition of Ceramic and Organoceramic Materials. Fundamental Aspects. Adv. Colloid Interface Sci..

[20] Mayr H., Ordung M., Ziegler G. (2006). Development of Thin Electrophoretically Deposited Hydroxyapatite Layers on TiAl6V4 Hip Prosthesis. J. Mater Sci..

[21] Wang C., Ma J., Cheng W., Zhang R. (2002). Thick Hydroxyapatite Coatings by Electrophoretic Deposition. Mater. Lett..

[22] Albrektsson T., Johansson C. (2001). Osteoinduction, Osteoconduction and Osseointegration. Eur. Spine J..

[23] Nikkhah M., Edalat F., Manoucheri S., Khademhosseini A. (2012). Engineering Microscale Topographies to Control the Cell-Substrate Interface. Biomaterials.

[24] Aplin A.E., Howe A.K., Juliano R.L. (1999). Cell Adhesion Molecules, Signal Transduction and Cell Growth. Curr. Opin. Cell Biol..

[25] Osak P., Łosiewicz B. (2018). EIS Study on Interfacial Properties of Passivated Nitinol Orthodontic Wire in Saliva Modified with Eludril^®^ Mouthwash. Prot. Met. Phys. Chem. Surf..

[26] Łosiewicz B., Osak P., Maszybrocka J., Kubisztal J., Stach S. (2020). Effect of Autoclaving Time on Corrosion Resistance of Sandblasted Ti G4 in Artificial Saliva Materials. Materials.

[27] Morawiec H., Goryczka T., Lelątko J., Lekston Z., Winiarski A., Rówiński E., Stergioudis G. (2010). Surface Structure of NiTi Alloy Passivated by Autoclaving. Mater. Sci. Forum.

[28] Łosiewicz B., Popczyk M., Goryczka T., Lelątko J., Smołka A., Kowalski P. (2013). Structure and Resistance to Electrochemical Corrosion of NiTi Alloy. Solid State Phenom..

[29] Zhitomirsky I., Gal-Or L. (1997). Electrophoretic Deposition of Hydroxyapatite. J. Mater Sci. Mater. Med..

[30] Ma J., Liang C.H., Kong L.B., Wang C. (2003). Colloidal Characterization and Electrophoretic Deposition of Hydroxyapatite on Titanium Substrate. J. Mater Sci. Mater. Med..

[31] Boccaccini A.R., Zhitomirsky I. (2002). Application of Electrophoretic and Electrolytic Deposition Techniques in Ceramics Processing. Curr. Opin. Solid State Mater. Sci..

[32] Yuehuei H.A., Draughn R.A. (1999). Mechanical Testing of Bone and the Bone-Implant Interface.

[33] Wei M., Ruys A.J., Swain M.V., Kim S.H., Milthorpe B.K., Sorrell C.C. (1999). Interfacial Bond Strength of Electrophoretically Deposited Hydroxyapatite Coatings on Metals. J. Mater Sci. Mater. Med..

[34] Dudek K., Dulski M., Goryczka T., Gerle A. (2018). Structural changes of hydroxyapatite coating electrophoretically deposited on NiTi shape memory alloy. Ceram. Int..

[35] Koutsopoulos S. (2002). Synthesis and Characterization of Hydroxyapatite Crystals: A Review Study on the Analytical Methods. J. Biomed. Mater. Res..

[36] Sinyayev V.A., Shustikova E.S., Griggs D., Dorofeev D.V. (2005). The Nature of P-O Bonds in the Precipitated Amorphous Calcium Phosphates and Calcium Magnesium Phosphates. Glass. Phys. Chem..

[37] Sauer G.R., Zunic W.B., Durig J.R., Wuthier R.E. (1994). Fourier Transform Raman Spectroscopy of Synthetic and Biological Calcium Phosphates. Calcif. Tissue Int..

[38] Elliott J.C. (2014). Structure and Chemistry of the Apatites and Other Calcium Orthophosphates.

[39] de Aza P.N., Guitián F., Santos C., de Aza S., Cuscó R., Artús L. (1997). Vibrational Properties of Calcium Phosphate Compounds. 2. Comparison between Hydroxyapatite and β-Tricalcium Phosphate. Chem. Mater..

[40] Cuscó R., Guitián F., de Aza S., Artús L. (1998). Differentiation between Hydroxyapatite and β-Tricalcium Phosphate by Means of μ-Raman Spectroscopy. J. Eur. Ceram. Soc..

